# Investigating Factors Associated With Spontaneous Remission in Individuals With Alcohol Use Disorder—Results From a Multi‐Site Longitudinal Cohort Study

**DOI:** 10.1111/adb.70153

**Published:** 2026-05-10

**Authors:** Judith Zaiser, Johannes Nitsche, Sabine Hoffmann, Sina Vetter, Nadja Samia Bahr, Clarissa Grundmann, Samanda Krasniqi, Fabian Arntz, Jens Strehle, Michael Marxen, Sabine Vollstädt‐Klein, Michael A. Rapp, Bernd Lenz, Rainer Spanagel, Michael Smolka, Andreas Heinz, Emanuel Schwarz, Falk Kiefer, Patrick Bach

**Affiliations:** ^1^ Department of Addictive Behavior and Addiction Medicine Central Institute of Mental Health, Medical Faculty Mannheim/Heidelberg University Mannheim/Heidelberg Germany; ^2^ German Center for Mental Health (DZPG), partner site Mannheim‐Heidelberg‐Ulm Mannheim/Heidelberg/Ulm Germany; ^3^ Hector Institute for Artificial Intelligence in Psychiatry Central Institute of Mental Health, Medical Faculty Mannheim/Heidelberg University Mannheim/Heidelberg Germany; ^4^ Department of Psychiatry and Psychotherapy Charité – Universitätsmedizin Berlin Berlin Germany; ^5^ Department of Psychosomatic Medicine Charité – Unversitätsmedizin Berlin Berlin Germany; ^6^ German Center for Mental Health (DZPG), partner site Berlin‐Potsdam Berlin/Potsdam Germany; ^7^ Department of Psychiatry and Psychotherapy Technische Universität Dresden Dresden Germany; ^8^ Department of Sports and Health Sciences University of Potsdam Potsdam Germany; ^9^ Center for Information Services and High Performance Computing (ZIH) Technische Universität Dresden Dresden Germany; ^10^ Mannheim Center for Translational Neurosciences (MCTN), Medical Faculty of Mannheim University of Heidelberg Mannheim Germany; ^11^ Social and Preventive Medicine, Department of Sports and Health Sciences, Intra‐Faculty Unit “Cognitive Sciences”, Faculty of Human Science, Faculty of Health Sciences Brandenburg, Research Area Services Research and e‐Health University of Potsdam Potsdam Germany; ^12^ Feuerlein Center on Translational Addiction Medicine (FCTS) Heidelberg University Heidelberg Germany; ^13^ Institute for Psychopharmacology Central Institute of Mental Health, Medical Faculty Mannheim/Heidelberg University Mannheim/Heidelberg Germany; ^14^ Section of Systems Neuroscience, Department of Psychiatry and Psychotherapy Technische Universität Dresden Dresden Germany; ^15^ Department of Psychiatry and Psychotherapy University of Tübingen Tübingen Germany; ^16^ German Center for Mental Health (DZPG), partner site Tübingen Tübingen Germany

**Keywords:** addiction, alcohol use disorder, AUD criteria, AUD remission, AUDIT, machine learning

## Abstract

Alcohol use disorder (AUD) is considered a chronic disorder with a highly variable course. Understanding this variability is crucial for identifying factors associated with persistence versus spontaneous remission. We analysed data from *N* = 462 individuals with AUD in an observational longitudinal cohort study to identify factors associated with spontaneous remission. All participants met Diagnostic and Statistical Manual of Mental Disorders, Fifth Edition (DSM‐5) criteria for AUD at study entry and were reassessed after 1 year, in which they were classified as being in spontaneous remission (*n* = 107), falling below the ≥ 2‐criteria threshold for AUD (*n* = 87) or continuing to meet AUD criteria (*n* = 296). Groups were compared on socio‐demographic, clinical and substance use variables at baseline and after 1 year. Additionally, we used machine learning models to identify baseline characteristics predicting a persistent course of AUD. Between‐group comparisons revealed that individuals who experienced spontaneous remission reported significantly lower AUD severity (*F*(2,459) = 25.17, *p* < 0.001), lower levels of alcohol intake (*F*(2,459) = 8.31, *p* = 0.013) and fewer drinking days (*F*(2,459) = 11.91, *p* < 0.001) at baseline and after 1 year. Machine learning analysis demonstrated moderate classification performance (AUC = 0.679), with the Alcohol Use Disorders Identification Test (AUDIT) sum score being the most informative predictor for group classification. Our findings indicate significant differences between spontaneously remitted and non‐remitted individuals on key alcohol‐related variables, supporting the clinical validity of remission as defined by the National Institute on Alcohol Abuse and Alcoholism (NIAAA). In addition, baseline characteristics may help identify individuals at risk of persistent AUD, enabling earlier identification of those who may benefit from specialized treatment.

**Trial Registration:**

DRKS number: DRKS00020580

## Graphical abstract

In a longitudinal cohort of individuals with alcohol use disorder (AUD), spontaneous remission after one year was associated with lower baseline AUD severity, lower levels of alcohol intake, and fewer drinking days. Machine learning showed moderate prediction of persistent AUD, with AUDIT score as the strongest predictor. Baseline characteristics serve as markers for AUD course and may help identify individuals at risk for a chronic course.

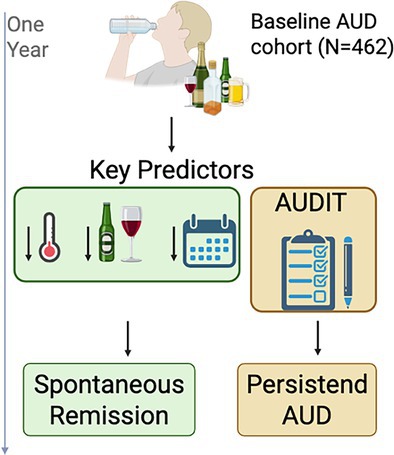

## 1 Introduction

Alcohol use disorder (AUD) is a chronic condition that causes substantial harm [1]. Nevertheless, the course of AUD varies substantially across individuals. Whereas some individuals exhibit increasing AUD severity over time, others show a stable course or even reductions in severity and remission [2, 3]. The National Institute on Alcohol Abuse and Alcoholism (NIAAA) defines remission from AUD as a state in which an individual has not met any Diagnostic and Statistical Manual of Mental Disorders, Fifth Edition (DSM‐5) criteria for AUD during the past 12 months, except alcohol craving [4, 5]. Previous research suggests that approximately half of individuals with AUD remit without specialized addiction treatment [3, 6], commonly referred to as spontaneous remission, whereas more than half require targeted treatment to attain remission [7, 8, 9]. Given this variability, it is important to understand which factors distinguish individuals who experience spontaneous remission from those who do not. This knowledge may help differentiate individuals who require active treatment from those for whom ongoing monitoring may be sufficient. To date, only a few studies have examined remission in the absence of specific treatment. One study investigating remission over a 3‐year period in *N* = 198 individuals with AUD identified older age, lower weekly alcohol consumption and absence of comorbid anxiety disorders as predictors of remission [10]. A 20‐year longitudinal study in *N* = 129 young male drinkers found that lower drinking frequency, lower AUD severity and higher educational level at a 5‐year follow‐up were associated with a greater likelihood of remission, whereas addiction treatment prior to the follow‐up was associated with a lower likelihood of remission [11]. Additionally, a 3‐year longitudinal study including *N* = 4078 individuals with AUD reported a median time to remission of 1.7 years and identified several factors associated with an increased likelihood of remission, including female gender, older age, Black or Latino ethnicity, presence of medical comorbidities, absence of comorbid drug use disorders, lower drinking levels and receiving addiction treatment prior to baseline [12]. Furthermore, a cross‐sectional study with *N* = 70 AUD individuals indicated that specific AUD criteria were differently associated with the course of AUD when comparing individuals who achieved remission with treatment to those who achieved remission without treatment [13].

Research on spontaneous remission in AUD remains limited. Identifying characteristics associated with spontaneous remission versus persistence of AUD may facilitate earlier detection of individuals at risk for persistent AUD and enable targeted interventions. Thus, the present study aims to examine whether the NIAAA definition of remission translates into clinically measurable differences between individuals who achieve spontaneous remission and those whose AUD persists over time. In addition, we applied a machine learning approach to identify baseline factors with high predictive value for spontaneous remission versus persistence of AUD.

This longitudinal cohort study enrolled individuals with AUD and followed them over a 1‐year period. Comprehensive data on socio‐demographic characteristics, clinical features and substance use patterns were collected at baseline and at the 1‐year reassessment to examine differences between individuals who achieved spontaneous remission and those who did not.

## 2 Methods and Materials

### 2.1 Study Sample

A total of 747 participants meeting DSM‐5 criteria for AUD were recruited into a multi‐site cohort study between February 2020 and July 2023 as part of a Collaborative Research Centre on AUD (TRR265; sites: Charité – Berlin University Medicine, Technical University Dresden and Central Institute of Mental Health Mannheim) [14]. Participants were recruited from the general population through flyers, newspaper advertisements and social media advertisements (Facebook). Eligibility criteria included a DSM‐5 diagnosis of mild to severe AUD within the past 12 months and no prior treatment for AUD.

Of the total sample, *N* = 462 participants completed the 1‐year follow‐up assessment and were included in the longitudinal analyses, whereas *n* = 285 participants did not complete the 1‐year follow‐up (see Figure S1 for the CONSORT study flow chart).

Inclusion criteria were (i) a diagnosis of AUD according to the DSM‐5, (ii) age between 16 and 65 years and (iii) the ability to understand study procedures and provide written informed consent.

Exclusion criteria included the following:

- use of medications affecting the central nervous system within the past 10 days or fewer than five drug half‐lives. Medications leading to exclusion included benzodiazepines and other sedatives or hypnotics, antipsychotic medication, antiepileptic medication and opioids; antidepressants and ADHD medications were permitted if participants had been on a stable dosage for at least 14 days and showed stable symptom control, with final eligibility determined by an experienced study physician;
- a diagnosis of bipolar disorder, psychotic disorder, schizophrenia or schizophrenic‐spectrum disorders or substance use disorders (except alcohol, nicotine and occasional cannabis use) according to DSM‐5;
- a history of severe head trauma;
- a history of central nervous system disorders (e.g., epilepsy, dementia, Parkinson's disease and multiple sclerosis);
- pregnancy or breastfeeding.

The presence of alcohol withdrawal symptoms within the past 12 months constituted an additional exclusion criterion for ethical reasons, in accordance with the ethics committee guidelines. Initiation of treatment for AUD during the 1‐year study period also resulted in exclusion from the study.

### 2.2 Study Design

This longitudinal cohort study consisted of four study visits: a baseline assessment followed by three follow‐up visits in 4‐month intervals. The present analyses focused on data from the baseline and 1‐year follow‐up visits, as the full set of variables relevant to the analyses was collected only at these two time points and not to the same extent at the intermediate follow‐ups. At both the baseline and 1‐year assessments, diagnostic criteria for AUD were evaluated according to the DSM‐5 by trained interviewers who received standardized rater training and were regularly supervised by psychiatrists. In addition, comprehensive socio‐demographic, mental health and substance use data were collected at these visits. Socio‐demographic data (see Table 1 for details) and the following questionnaires were administered: DSM‐5 AUD criteria, Alcohol Use Disorders Identification Test (AUDIT), Perceived Stress Scale (PSS), General Depression Scale (ADS), Brief Symptom Inventory (BSI), Childhood Trauma Screener (CTS), State–Trait Anxiety Inventory (STAI trait) and Alcohol Quantity‐Frequency measures [15] (see Table S1 for the full list of variables and Table S2 for details on the questionnaires). The BSI was used to assess psychological distress, with lower scores indicating lower symptom burden.

**TABLE 1:** Demographic data, alcohol use and severity measures for all three groups at baseline.

Subgroup								
	AUD (*n* = 269)	Subthreshold AUD (*n* = 86)	Remission (*n* = 107)	Statistics	Significance	Post hoc		
Demographical variables						AUD vs. subthr. AUD	AUD vs. remission	Subthr. AUD vs. remission
Gender (female; male)	95; 174	37; 49	44; 63	*χ* ^2^ = 2.18	*p* = 0.336			
Age (years)	37.56 (12.36)	39.84 (14.09)	35.63 (12.29)	*F* = 2.63	*p* = 0.073			
Family status (living alone or not)	149; 115	45; 38	71; 33	*χ* ^2^ = 5.17	*p* = 0.075			
Own children (yes; no)	90; 174	35; 48	25; 79	*χ* ^2^ = 7.03	***p* = 0.030**	*p* = 0.515	*p* = 0.194	***p* = 0.027**
Migration background (yes; no)	12; 256	2; 84	5; 102	*Z* = 0.76	*p* = 0.727			
Job, last 3 months (yes; no)	211; 53	69; 14	84; 20	*χ* ^2^ = 0.42	*p* = 0.811			
Income (< €2000; ≥ €2000)	140; 124	46; 37	56; 48	*χ* ^2^ = 0.15	*p* = 0.929			
Substance use patterns								
AUD criteria	4.48 (1.67)	3.4 (1.18)	3.46 (1.59)	*F* = 25.17	***p* < 0.001**	***p* < 0.001**	***p* < 0.001**	*p* = 1.000
AUDIT	16.32 (4.8)	12.41 (4.65)	12.43 (4.81)	*F* = 35.82	***p* < 0.001**	***p* < 0.001**	***p* < 0.001**	*p* = 1.000
Comorbid substance use (none; use of at least one further substance)	224; 44	83; 3	98; 9	*χ* ^2^ = 12.02	***p* = 0.002**	***p* = 0.004**	*p* = 0.093	*p* = 0.882
Smoking (yes; no)	127; 131	25; 58	38; 64	*χ* ^2^ = 11.08	***p* = 0.004**	***p* = 0.006**	*p* = 0.112	*p* = 0.977
Cannabis use last 3 months (yes; no)	76; 193	14; 72	26; 81	*χ* ^2^ = 5.02	*p* = 0.081			
Clinical scales								
PSS	16.44 (6.68)	14.11 (5.83)	14.79 (6.36)	*F* = 5.13	***p* = 0.006**	***p* = 0.014**	*p* = 0.094	*p* = 1.000
ADS	11.28 (7.3)	8.25 (5.22)	10.13 (7.74)	*F* = 5.9	***p* = 0.003**	***p* = 0.002**	*p* = 0.487	*p* = 0.219
BSI	23.11 (19.08)	16.93 (14.06)	18.01 (18.27)	*F* = 5.22	***p* = 0.006**	***p* = 0.021**	***p* = 0.049**	*p* = 1.000
STAI trait	40.28 (9.89)	36.06 (8.04)	37.57 (10.5)	*F* = 7.16	***p* < 0.001**	***p* = 0.002**	*p* = 0.050	*p* = 0.879
CTS	7.68 (2.84)	7.44 (2.43)	7.64 (2.84)	*F* = 0.25	*p* = 0.783			
Comorbid mental illness (healthy; at least one more mental disorder)	191; 78	64; 22	80; 27	*χ* ^2^ = 0.74	*p* = 0.692			
Alcohol drinking								
Last 3 months (g alcohol/day)	6.73 (3.99)	5.14 (2.49)	5.55 (3.35)	*F* = 8.31	***p* < 0.001**	***p* = 0.001**	***p* = 0.013**	*p* = 1.000
Typical weekday (g alcohol/day)	4.75 (3.72)	3.4 (1.83)	3.53 (2.91)	*F* = 8.64	***p* < 0.001**	***p* = 0.003**	***p* = 0.004**	*p* = 1.000
Typical weekend (g alcohol/day)	8.35 (4.61)	6.55 (3.38)	6.82 (3.76)	*F* = 8.63	***p* < 0.001**	***p* = 0.002**	***p* = 0.005**	*p* = 1.000
Last drinking day (g alcohol)	6.69 (5.01)	5.54 (4.64)	4.92 (3.76)	*F* = 6.14	***p* = 0.002**	*p* = 0.145	***p* = 0.003**	*p* = 1.000
Drinking days (%)	59.52 (26.71)	58.16 (24.43)	45.25 (25.34)	*F* = 11.91	***p* < 0.001**	*p* = 1.000	***p* < 0.001**	***p* = 0.002**
Heavy drinking days (%)	25.34 (25.66)	15.97 (20.49)	16.27 (20.09)	*F* = 8.57	***p* < 0.001**	***p* = 0.004**	***p* = 0.002**	*p* = 1.000

*Note:* Income: with regard to the mean German net income; gender: none of the participants assigned themselves to ‘divers’; all variables were received to T0; continuous variables: univariate analyses of variance with *F*‐tests, mean (SD); categorical variables: chi‐square (*χ* ^2^) test or Fisher's exact test, *Z*. *p* < 0.05 indicated in bold.

Abbreviations: ADS = General Depression Scale (German: Allgemeine Depressionsskala); AUD criteria = Alcohol Use Disorder diagnostic criteria; AUDIT = Alcohol Use Disorders Identification Test; BSI = Brief Symptom Inventory; CTS = Childhood Trauma Screener; PSS = Perceived Stress Scale; STAI trait = State–Trait Anxiety Inventory.

Participants received monetary compensation for their participation.

### 2.3 Statistical Analyses

Analyses included data from participants whose AUD diagnostic criteria were available at both baseline and the 1‐year follow‐up. Missing data were not imputed because the limited number of assessment time points and the risk of bias rendered imputation inappropriate. A total of *N* = 285 participants discontinued study participation. Although individuals reported various reasons for discontinuation, comparisons between participants who discontinued and those who completed the study revealed no significant differences for the majority of baseline variables, including age, gender and baseline AUD symptom count (Table S3). Reported reasons for study discontinuation included loss of interest, extended time burden, insufficient monetary compensation and the need for qualified treatment (see Supporting Information for details on dropout reasons and comparisons between completers and non‐completers, including Little's MCAR test results).

Participants were categorized into three groups based on AUD symptom status at the 1‐year assessment: (i) individuals meeting the NIAAA definition of remission (i.e., meeting no AUD criteria, with the exception of craving), (ii) individuals who continued to meet criteria for AUD (i.e., ≥ 2 AUD criteria) and (iii) individuals who met more than zero AUD criteria but fewer than two AUD criteria (i.e., one criterion other than craving), hereafter referred to as the *subthreshold* AUD group.

Given the timing of the follow‐up assessments, individuals meeting criteria for remission at the 1‐year follow‐up were classified as being in early remission, corresponding to an absence of AUD criteria for a period of 3–12 months.

To examine whether outcome groups differed in other characteristics, we compared socio‐demographic variables, clinical measures and substance use patterns at baseline and at the 1‐year follow‐up (see Table S1 for a complete list of variables). Selection of variables was guided by prior literature on remission in AUD and evidence supporting the relevance of specific factors [10, 11, 12, 13]. Between‐group differences at baseline and follow‐up were assessed using univariate analyses of variance (ANOVAs) and chi‐square tests. To account for potential baseline differences, follow‐up comparisons were additionally conducted using analyses of covariance (ANCOVAs), with baseline values of the respective variables entered as covariates. Comparisons between participants who discontinued the study and those who completed the 1‐year follow‐up were conducted at baseline using Student's *t*‐tests and chi‐square tests. All statistical analyses were performed using the Statistical Package for the Social Sciences software (SPSS, IBM Corp., Somers, NY, USA), version 29.0.

#### 2.3.1 Classification of Remission Versus Persistence of AUD

To determine which baseline characteristics contributed most to classifying individuals into the prospective outcome groups of remission, persistent AUD and subthreshold AUD, we applied a machine learning approach using a random forest (RF) classifier and an extensive set of baseline variables. Based on previous findings, we included a broad range of factors previously shown to influence the course of AUD [10, 11, 12, 13], encompassing AUD severity and individual AUD criteria, clinical scales and substance use patterns. The RF model incorporated 37 baseline variables (Table S1) as potential predictors for differentiating between the three outcome groups. The statistical significance of model performance was evaluated using a permutation‐testing framework to determine whether predictive accuracy exceeded chance levels. Feature relevance was first assessed using Gini feature importance, an intrinsic measure of feature importance in RF models. Subsequently, Shapley Additive Explanations (SHAP) values were computed to provide insight into the overall importance of features across the model (global interpretability) and the contribution of each feature to individual predictions (local interpretability), whereas Gini importance only reflects global feature importance. This approach allowed us to interpret both the relative importance of individual predictors and the specific outcome group towards which they contributed. (Supporting Information provides further details on the model specifications, an additional SHAP analysis stratified by age and consistency testing of the RF model).

#### 2.3.2 The 10‐Fold Cross‐Validation (CV) Method

Tenfold CV was used to evaluate the performance of the RF classifier. The dataset was randomly partitioned into 10 approximately equal folds. In each iteration, nine folds were used for model training and the remaining fold for validation, such that each fold served once as the validation set. Performance metrics were averaged across all folds to obtain a robust estimate of model generalization and to reduce variance attributable to data partitioning.

### 2.4 Assessment of Potential Clinical Utility of the AUDIT Score to Identify Individuals With Persistent AUD

To investigate whether a cut‐off value could be identified for the most informative baseline factor selected by the RF model to distinguish individuals with persistent AUD from those without persistent AUD, we constructed receiver operating characteristic (ROC) curves and calculated the area under the curve (AUC). For this analysis, individuals with persistent AUD were compared with all other participants (i.e., those with subthreshold AUD or remission). The optimal threshold was determined using the Youden index, which maximizes the combined sensitivity and specificity, making it useful for selecting the cut‐off value with the highest discriminative power (see Supporting Information Results for the results of AUC and ROC).

## 3 Results

**FIGURE 1:**
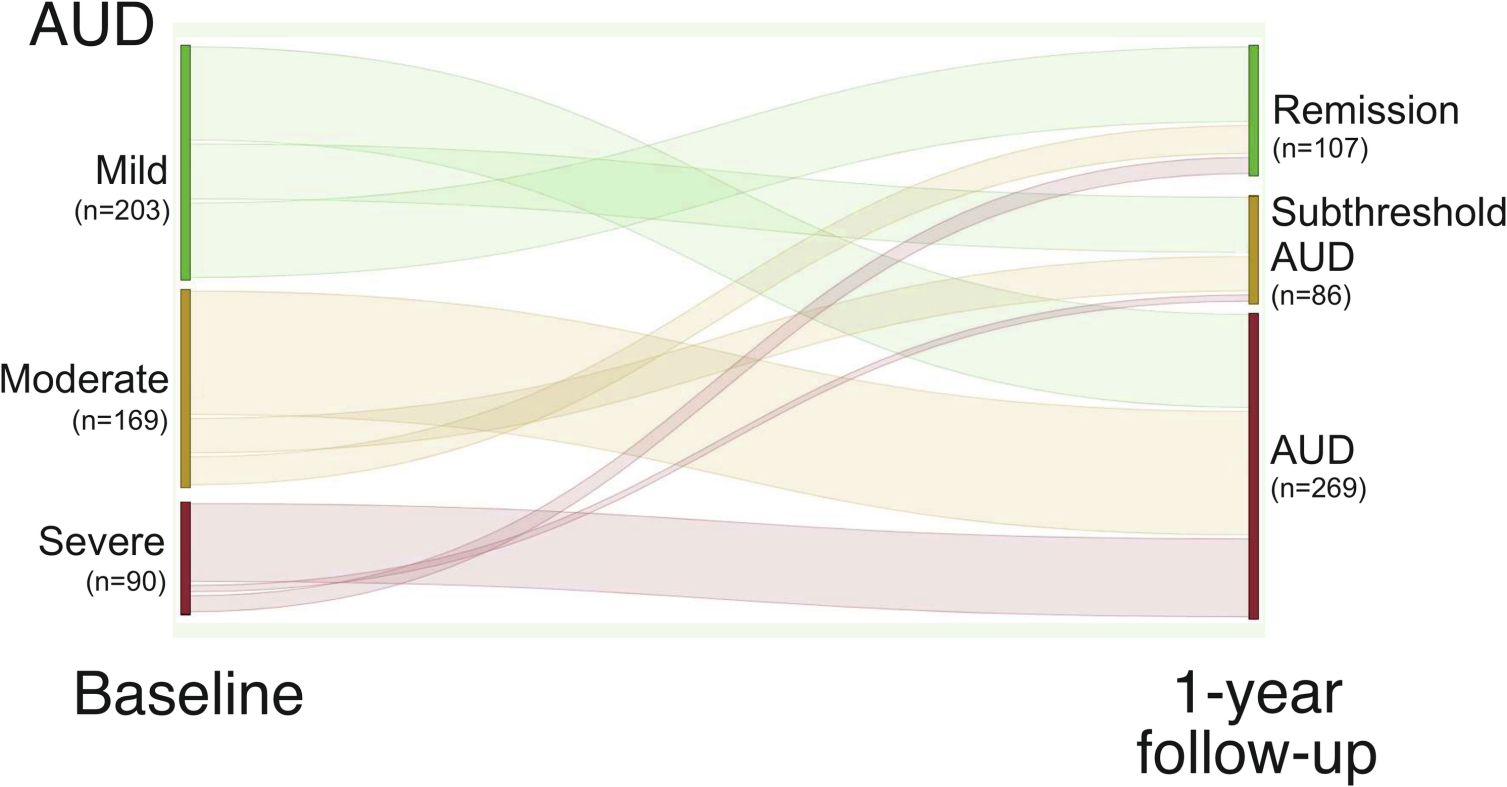
Sankey plot depicting the AUD course over the year. *N* = 462 AUD individuals with different severity levels (mild, moderate and severe) were enrolled at baseline. After 1 year, *n* = 269 individuals were still identified with AUD, *n* = 107 achieved remission and *n* = 86 still met the criteria of AUD, but below the diagnostic cut‐off and hence were considered individuals with subthreshold AUD.

At baseline, all *N* = 462 participants met criteria for AUD, with a mean symptom count of 4.04 (SD = 1.65). At the 12‐month follow‐up, the mean reduction in AUD criteria was 1.82 (SD = 1.92). A total of *n* = 269 participants (58.23%) continued to meet criteria for AUD, whereas *n* = 107 participants (23.16%) met the NIAAA definition of early remission without having received treatment and were therefore classified as experiencing spontaneous remission. The remaining *n* = 86 participants (18.61%) met one AUD criterion at follow‐up, falling below the diagnostic threshold for AUD and thus classified as having subthreshold AUD (Figure 1).

### 3.1 Group Differences at Baseline

Baseline comparisons across the three outcome groups revealed significant differences in AUD symptom count (*F*(2,459) = 25.17, *p* < 0.001), AUDIT scores (*F*(2,438) = 35.82, *p* < 0.001), alcohol intake during the 3 months prior to baseline (*F*(2,459) = 8.31, *p* < 0.001), percentage of drinking days (*F*(2,459) = 11.91, *p* < 0.001), percentage of heavy drinking days (*F*(2,459) = 8.57, *p* < 0.001), comorbid substance use (*χ* ^2^(2, *N* = 461) = 12.02, *p* = 0.002), smoking status (*χ* ^2^(2, *N* = 443) = 11.08, *p* = 0.004), PSS scores (*F*(2,435) = 5.13, *p* = 0.006), ADS (*F*(2,441) = 5.9, *p* = 0.003), anxiety (*F*(2,448) = 7.16, *p* < 0.001) and BSI scores (*F*(2,438) = 5.22, *p* = 0.006) (Table 1).

Post hoc analyses with Bonferroni correction showed that, at baseline, individuals who achieved early remission after the 1‐year period exhibited lower AUD symptom counts (mean ± SD: 3.46 ± 1.59 vs. 4.48 ± 1.67; *p*_Remission vs. AUD_ < 0.001), lower AUDIT scores (12.43 ± 4.81 vs. 16.32 ± 4.8; *p*_Remission vs. AUD_ < 0.001) and lower BSI scores (18.01 ± 18.27 vs. 23.11 ± 19.08; *p*_Remission vs. AUD_ = 0.049) at baseline, compared with individuals with persistent AUD. Furthermore, the early remission group reported lower alcohol intake during the 3 months prior to baseline (5.55 ± 3.35 vs. 6.73 ± 3.99; *p*_Remission vs. AUD_ < 0.013), a lower percentage of drinking days (45.25 ± 25.34 vs. 59.52 ± 26.71; *p*_Remission vs. AUD_ < 0.001) and a lower percentage of heavy drinking days (16.27 ± 20.09 vs. 25.34 ± 25.66; *p*_Remission vs. AUD_ = 0.002).

When comparing individuals who later achieved early remission with those classified as having subthreshold AUD (i.e., meeting exactly one AUD criterion), few baseline differences were observed. The only significant difference was a lower percentage of drinking days prior to baseline in the remission group (45.25 ± 25.34 vs. 58.16 ± 24.43; *p*_Remission vs. Subthreshold AUD_ = 0.002) (see Table 1). Significant differences between the subthreshold AUD and persistent AUD groups were observed at baseline and at follow‐up (see Supporting Information for details).

### 3.2 Group Differences at the End of the Year Including Controlling for Baseline Differences

Next, group differences at the 1‐year follow‐up were examined while controlling for baseline values. Comparisons across the three outcome groups revealed significant differences in AUD symptom count (*F*(2,458) = 278.47, *p* < 0.001), AUDIT scores (*F*(2,382) = 31.16, *p* < 0.001), percentage of drinking days (*F*(2,458) = 6.42, *p* = 0.002), percentage of heavy drinking days (*F*(2,457) = 12.7, *p* < 0.001) and BSI scores (*F*(2,379) = 3.67, *p* = 0.026) (see Table 2 for details).

**TABLE 2:** Analyses of covariance to identify changes after 1 year in alcohol use and severity measures in consideration of baseline values.

Subgroup								
	AUD (*n* = 269)	Subthreshold AUD (*n* = 86)	Remission (*n* = 107)	Statistics	Significance	Post hoc		
Substance use patterns						AUD vs. subthr. AUD	AUD vs. remission	Subthr. AUD vs. remission
AUD criteria	3.47 (1.64)	1 (0)	0.07 (0.25)	*F* = 278.47	***p* < 0.001**	***p* < 0.001**	***p* < 0.001**	***p* < 0.001**
AUDIT	15.08 (5.16)	10.68 (5.12)	9.07 (4.13)	*F* = 31.16	***p* < 0.001**	***p* < 0.001**	***p* < 0.001**	***p* = 0.016**
Clinical scales								
PSS	16.74 (6.86)	13.83 (5.58)	15.04 (7.93)	*F* = 1.66	*p* = 0.192			
ADS	13.48 (8.93)	9.58 (6.63)	11.34 (9.24)	*F* = 2.55	*p* = 0.080			
BSI	27.24 (24.6)	16.09 (16.01)	19.16 (23.85)	*F* = 3.67	***p* = 0.026**	***p* = 0.049**	*p* = 0.183	*p* = 1.000
Alcohol drinking								
Drinking days (%)	55.61 (29.48)	49.32 (28.16)	36.61 (26.99)	*F* = 6.42	***p* = 0.002**	*p* = 0.141	***p* = 0.002**	*p* = 0.912
Heavy drinking days (%)	21.43 (23.59)	9.41 (16.92)	8.56 (11.2)	*F* = 12.7	***p* < 0.001**	***p* = 0.001**	***p* < 0.001**	*p* = 1.000

*Note:* Univariate analyses of variance with *F*‐tests, mean (SD). *p* < 0.05 indicated in bold.

Abbreviations: ADS = Allgemeine Depressionsskala; AUD criteria = Alcohol Use Disorder criteria count (0–11); AUDIT = Alcohol Use Disorders Identification Test; BSI = Brief Symptom Inventory; PSS = Perceived Stress Scale.

Post hoc analyses with Bonferroni correction indicated that remitted individuals had significantly lower AUD symptom counts (estimated mean ± SE: 0.23 ± 0.12 vs. 3.35 ± 0.07; *p*_Remission vs. AUD_ < 0.001) and lower AUDIT scores (10.27 ± 0.41 vs. 14.13 ± 0.27; *p*_Remission vs. AUD_ < 0.001) at the 1‐year follow‐up, compared with individuals with persistent AUD. Further, the remission group exhibited a lower percentage of heavy drinking days (11.2 ± 1.58 vs. 19.47 ± 0.99; *p*_Remission vs. AUD_ < 0.001) and a lower percentage of drinking days (44.46 ± 2.12 vs. 53 ± 1.32; *p*_Remission vs. AUD_ = 0.002). Comparisons between the remission and subthreshold AUD groups showed that, after controlling for baseline values, remitted individuals had lower AUD symptom counts (0.23 ± 0.12 vs. 1.18 ± 0.13; *p*_Remission vs. Subthreshold AUD_ < 0.001) and lower AUDIT scores (10.27 ± 0.41 vs. 11.94 ± 0.44; *p*_Remission vs. Subthreshold AUD_ = 0.016) at the 1‐year follow‐up (see Table 2).

### 3.3 Group Differences at the End of the Year

Lastly, group differences in AUD severity, clinical measures and substance use variables were analysed at the 1‐year follow‐up. Comparisons across the three outcome groups showed significant differences in AUD symptom count (*F*(2,459) = 328.28, *p* < 0.001), AUDIT scores (*F*(2,401) = 62.73, *p* < 0.001), percentage of drinking days (*F*(2,459) = 16.82, *p* < 0.001), percentage of heavy drinking days (*F*(2,458) = 21.48, *p* < 0.001), PSS scores (*F*(2,400) = 5.72, *p* = 0.004), ADS (*F*(2,399) = 6.24, *p* = 0.002) and BSI scores (*F*(2,398) = 8.03, *p* < 0.001) (see Table 3 for details).

**TABLE 3:** Univariate analyses of variance to identify differences between the three groups after 1 year in alcohol use, severity measures and clinical scales.

Subgroup								
	AUD (*n* = 269)	Subthreshold AUD (*n* = 86)	Remission (*n* = 107)	Statistics	Significance	Post hoc		
Substance use patterns						AUD vs. subthr. AUD	AUD vs. remission	Subthr. AUD vs. remission
AUD criteria	3.47 (1.64)	1 (0)	0.07 (0.25)	*F* = 328.28	***p* < 0.001**	***p* < 0.001**	***p* < 0.001**	***p* < 0.001**
AUDIT	15.22 (5.11)	10.6 (5.05)	9.05 (4.12)	*F* = 62.73	***p* < 0.001**	***p* < 0.001**	***p* < 0.001**	*p* = 0.116
Clinical scales								
PSS	16.72 (6.88)	13.83 (5.57)	15.21 (7.84)	*F* = 5.72	***p* = 0.004**	***p* = 0.004**	*p* = 0.218	*p* = 0.553
ADS	13.44 (8.85)	9.68 (6.62)	11.52 (9.13)	*F* = 6.24	***p* = 0.002**	***p* = 0.002**	*p* = 0.194	*p* = 0.466
BSI	27.16 (24.31)	16.2 (16.12)	20.07 (24.05)	*F* = 8.03	***p* < 0.001**	***p* = 0.001**	***p* = 0.035**	*p* = 0.794
Alcohol drinking								
Drinking days (%)	55.61 (29.48)	49.32 (28.16)	36.61 (26.99)	*F* = 16.82	***p* < 0.001**	*p* = 0.232	***p* < 0.001**	***p* = 0.007**
Heavy drinking days (%)	21.43 (23.59)	9.41 (16.92)	8.56 (11.2)	*F* = 21.48	***p* < 0.001**	***p* = 0.001**	***p* < 0.001**	*p* = 1.000

*Note:* Univariate analyses of variance with *F*‐tests, mean (SD). *p* < 0.05 indicated in bold.

Abbreviations: ADS = Allgemeine Depressionsskala; AUD criteria = Alcohol Use Disorder criteria count (0–11); AUDIT = Alcohol Use Disorders Identification Test; BSI = Brief Symptom Inventory; PSS = Perceived Stress Scale.

**FIGURE 2:**
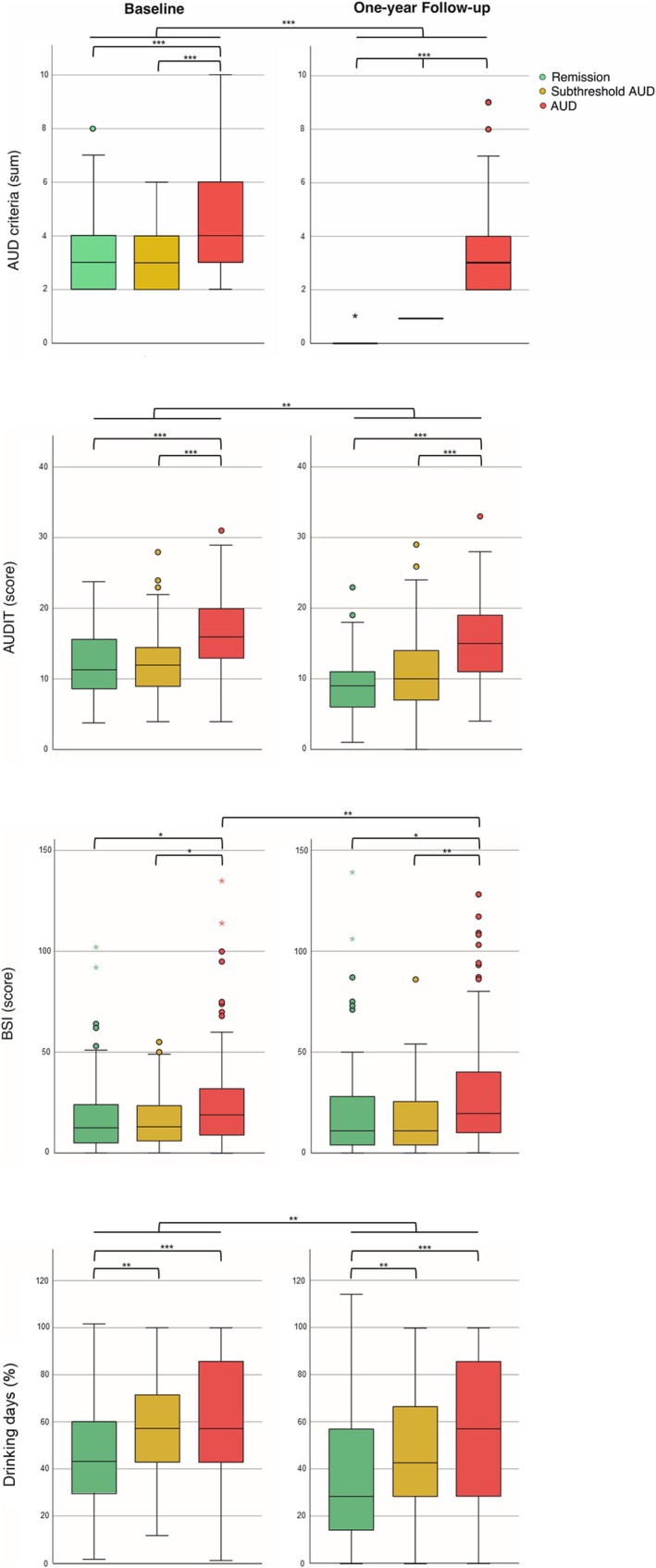
The presented boxplots depict the results of group comparisons (remission, subthreshold AUD and AUD) at the two time points—baseline and 1‐year follow‐up—for the sum of AUD criteria, AUDIT score, BSI score and drinking days (percentage). Significant differences between the groups within each time point, as well as within the groups across time points, are indicated. ***p < 0.001; **p < 0.01; *p < 0.05.

Post hoc analyses with Bonferroni correction demonstrated that, at the 1‐year follow‐up, individuals in remission exhibited lower AUD symptom counts (mean ± SD: 0.07 ± 0.25 vs. 3.47 ± 1.64; *p*_Remission vs. AUD_ < 0.001), lower AUDIT scores (9.05 ± 4.12 vs. 15.23 ± 5.21; *p*_Remission vs. AUD_ < 0.001), lower percentages of heavy drinking days (8.56 ± 11.2 vs. 21.43 ± 23.59; *p*_Remission vs. AUD_ < 0.001) and drinking days (36.61 ± 26.99 vs. 55.61 ± 29.48; *p*_Remission vs. AUD_ < 0.001) and significantly lower BSI scores (20.07 ± 24.05 vs. 27.16 ± 24.31; *p*_Remission vs. AUD_ = 0.035) compared with individuals with persistent AUD. Comparisons between the remission and subthreshold AUD groups indicated that remitted individuals had lower AUD symptom counts (0.07 ± 0.25 vs. 1 ± 0; *p*_Remission vs. Subthreshold AUD_ < 0.001) and a lower percentage of drinking days (36.61 ± 26.99 vs. 49.32 ± 28.16; *p*_Remission vs. Subthreshold AUD_ = 0.007) at the 1‐year follow‐up (see Table 3 and Figure 2).

### 3.4 Classification of Remission Versus Subthreshold AUD Versus Persistence of AUD

The RF classifier achieved an average test AUC of 0.68 ± 0.06 (mean ± SD) across the 10 CV folds. In contrast, the empirical null distribution of average test AUCs had a mean of 0.501, indicating that the model performance was significantly better than chance (*p* = 0.002) (see Figures S2 and S3).

**FIGURE 3:**
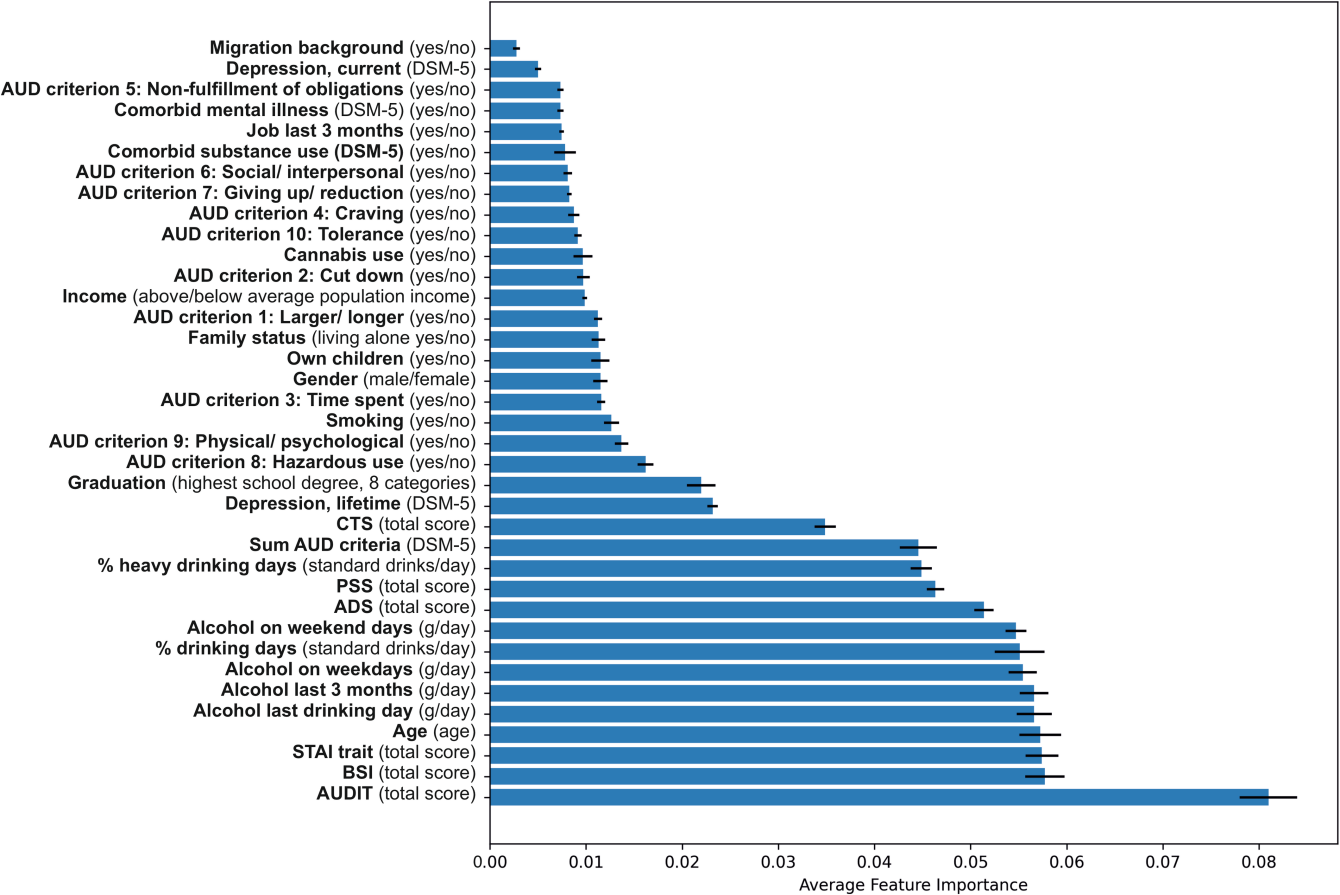
Average Gini feature importance of the 37 baseline values in the RF model. Features with larger values (i.e., with longer bars) on the *x*‐axis (e.g., AUDIT) have a higher Gini importance, indicating that they are more influential in the classification process. Conversely, features closer to the *y*‐axis (e.g., migration background) have a lower Gini importance and contribute less to class differentiation. Error bars represent the standard deviation of feature importance across multiple model iterations.

Analysis of Gini feature importance identified the AUDIT score, BSI and STAI trait scores as the most influential predictors contributing to group classification by minimizing Gini impurity (Figure 3; see Supporting Information for details on questionnaires). Consistent with these findings, the SHAP analysis confirmed the AUDIT score as the most relevant predictor and further highlighted the percentage of drinking days as a highly informative factor. Comparable performance across additional classification models supported the consistency of the RF classifier (see Supporting Information Results).

## 4 Discussion

Results from this longitudinal multi‐site cohort study indicate that approximately one quarter of the study cohort (23%), composed primarily of individuals with mild to moderate AUD, met criteria for remission after 1 year in the absence of specific addiction treatment. Pronounced reductions over the 1‐year period in the percentage of drinking and heavy drinking days, as well as reductions in psychological distress among individuals who achieved spontaneous remission compared with those who continued to meet AUD criteria, further support the clinical validity and relevance of the remission definition proposed by the NIAAA. Individuals who achieved spontaneous remission at the 1‐year follow‐up differed significantly at baseline from those with persistent AUD with respect to alcohol use patterns, AUD severity and psychological distress. These findings indicate that readily available baseline clinical characteristics may help identify individuals who are likely to experience spontaneous remission. This interpretation was also supported by machine learning analyses, which demonstrated that baseline clinical features could prospectively classify individuals who continued to meet criteria for AUD versus those who no longer met diagnostic criteria, including those in spontaneous remission and those with subthreshold AUD. Among the evaluated predictors, the AUDIT score emerged as the most informative single clinical feature. Baseline AUDIT scores achieved a combined sensitivity and specificity of 85% and 49% for identifying individuals with a persistent course of AUD compared with those who achieved remission or subthreshold status. Taken together, these findings substantiate the clinical validity of the NIAAA definition of remission and highlight that individuals with persistent versus spontaneously remitting AUD differ in their clinical profiles. Consideration of these baseline features may aid in recognizing individuals at risk for a chronic course of AUD and help inform decisions regarding the need for targeted addiction treatment.

The presented findings on spontaneous remission in AUD align with previous research indicating that individuals with AUD remit without receiving specialized addiction treatment [3, 16]. The remission rates observed in this study are consistent with recent reports [3, 6]. Although all participants met diagnostic criteria for AUD at baseline in the current study, identifying baseline characteristics that differentiate individuals who later remit spontaneously from those with persistent or subthreshold AUD remains clinically relevant. Understanding such characteristics may provide valuable insight into individual risk profiles and trajectories of AUD over time.

Our analyses examined key baseline predictors differentiating spontaneous remission from non‐remission outcomes, including persistent AUD and subthreshold AUD. Although we defined spontaneous remission as occurring without targeted treatment, we cannot rule out that mere observation of participants' drinking behaviour during the study may have influenced the outcome. Group comparisons suggest that individuals who achieved spontaneous remission at the 1‐year follow‐up already showed a lower AUD severity and lower levels of alcohol use at baseline, which remained evident at follow‐up, including controlling for baseline differences. Moreover, spontaneously remitted individuals demonstrated significantly lower AUD severity, reduced alcohol consumption and lower psychological distress at the 1‐year assessment. These findings highlight two important points. First, the remission definition proposed by the NIAAA translates into significant differences across clinically relevant outcomes, such as alcohol use patterns and psychological distress, which are closely linked to long‐term mental and physical health. This supports the ecological validity and clinical usefulness of the NIAAA definition of remission. Second, the findings indicate that individuals who meet the criteria for AUD at baseline can follow different courses over time, depending on variability in individual clinical features. In this context, our results suggest that beyond diagnostic criteria, the AUDIT score and alcohol use indices provide important information for identifying individuals who remit spontaneously from those who continue to meet criteria for AUD or remain in a subthreshold state. The importance of alcohol use patterns for the subsequent course of AUD has been consistently reported in prior research. For example, a 3‐year longitudinal study linked remission to a reduction of six drinks per week and found that higher baseline alcohol consumption was associated with an increased risk of persistence [10]. Similarly, a 20‐year study demonstrated that lower drinking frequency at baseline predicted remission [11]. Additional studies have shown that lower alcohol consumption and fewer heavy drinking days are associated with a greater likelihood of remission [10, 17, 18]. Together with these findings, our results reinforce the central role of (heavy) drinking behaviour in AUD progression. Furthermore, current alcohol consumption has been shown to predict relapse risk and may therefore serve as a useful reference point for relapse prevention strategies [19]. In contrast to some previous studies, age, gender, educational level and anxiety disorders did not emerge as significant predictors of spontaneous remission in the present sample [10, 11, 12]. Several methodological differences may account for these discrepancies. Previous studies varied considerably in sample compositions (e.g., population‐based surveys, male‐only cohorts or primary care samples), follow‐up duration (ranging from 3 years to several decades) and definitions of remission (e.g., sustained diagnostic remission or remission from unhealthy drinking). By contrast, the present study focused on remission over a comparatively short follow‐up period of 1 year. This suggests that over shorter time frames, differences in drinking quantity and frequency may become apparent earlier, whereas socio‐demographic and psychiatric factors such as age, education, gender and anxiety disorders may exert a stronger influence on longer term AUD trajectories. In line with this interpretation, a study with a 9‐year follow‐up identified physical activity as an additional factor whose reduction was associated with AUD development [20].

Although the aim of the study was to identify baseline predictors differentiating spontaneous remission from persistent AUD, not all individuals could be clearly assigned to one of these two groups. A subset of participants fell below the diagnostic threshold of two AUD criteria at follow‐up but did not meet criteria for remission and were therefore classified as having subthreshold AUD. Clinically, this group may represent individuals with partial improvement who no longer meet full AUD criteria but continue to exhibit residual symptoms. They exhibited higher alcohol use and greater AUD severity at the 1‐year follow‐up than remitted individuals. This pattern highlights the relevance of alcohol consumption as a key factor in spontaneous remission and suggests that they occupy an intermediate position on the recovery continuum rather than being nearly remitted. Analytically, distinguishing this group from both persistent AUD and full remission may provide a more nuanced representation of recovery trajectories and avoid potential misclassification. Future research should further examine whether these individuals should remain as a separate group or be assigned to either the remission or the AUD group.

Machine learning analyses incorporating 37 baseline clinical variables showed sufficient predictive performance. Both Gini importance and SHAP analyses consistently identified AUDIT, assessed at baseline when all participants met criteria for AUD, as the most informative predictor distinguishing remission from non‐remission outcomes (i.e., subthreshold AUD and persistent AUD). An advantage of this approach is its ability to rank the relative contribution of correlated predictors, thereby providing insight into their differential relevance. From a clinical perspective, these findings reinforce the value of the AUDIT as a screening instrument for risky drinking and as a diagnostic marker of AUD severity. Given that the AUDIT captures both drinking frequency and quantity, its predictive utility likely reflects its comprehensive assessment of alcohol consumption patterns. Consistent with prior work, previous studies have found that the AUDIT is useful for AUD prognosis, including the prediction of relapse following treatment [17]. Our analyses further indicate that an AUDIT score greater than 11, commonly interpreted as reflecting hazardous or harmful alcohol use (score 8–14), identifies individuals who are less likely to remit spontaneously, with high sensitivity and moderate specificity.

Clinically, this suggests that individuals with AUDIT scores above 11 may benefit from closer monitoring, as their likelihood of spontaneous remission appears reduced compared with individuals scoring 11 or below. These findings may also have implications for AUD treatment conceptualization. The transition from the DSM‐IV distinction between ‘abuse’ and ‘dependence’ to the single DSM‐5 diagnosis of AUD has generated debate, with concerns that a unified diagnostic category may obscure important differences in severity and treatment needs [21]. Our results highlight the importance of considering individual symptom severity and risk profiles, rather than relying solely on a broad diagnostic label, to optimize treatment planning and the allocation of specialized addiction treatment.

Beyond the AUDIT, additional alcohol‐related variables contributed meaningfully to outcome differentiation. In particular, the percentage of drinking days at baseline emerged as a key indicator distinguishing remission from non‐remission outcomes, consistent with earlier analyses in this study and prior literature emphasizing drinking patterns as prognostic markers for AUD trajectories towards remission [10, 11, 12]. Together, these findings reinforce the relevance of alcohol consumption behaviour in shaping the course of AUD.

Finally, psychosocial factors, including psychological distress, trait anxiety and age, also contributed relevant information, in line with previous research. In contrast, individual AUD criteria were relevant but had lower predictive importance, as demonstrated in the machine learning models. This aligns with previous studies showing that individual AUD criteria differ in their prognostic relevance for severe AUD progression and that their importance varies by age and overall severity [21, 22, 23]. Overall, our findings suggest that whereas individual AUD criteria provide useful diagnostic information, broader clinical and behavioural features play a more prominent role in distinguishing spontaneous remission from persistent AUD.

### 4.1 Limitations

This study presents data from a well‐characterized and sizable cohort of individuals with AUD. However, the sample predominantly comprised individuals with mild to moderate AUD, which may limit the generalizability of the findings to individuals with severe AUD, from whom previous studies have reported a more progressive course of AUD severity [24]. Additionally, the exclusion of individuals taking certain CNS‐active medications may have introduced further selection into the sample, potentially leading to an underrepresentation of individuals with greater psychiatric or clinical complexity and thereby further limiting the generalizability of the findings to broader AUD populations. Although the 1‐year follow‐up period was sufficient to detect early spontaneous remission, longer follow‐up durations are needed to assess sustained remission and long‐term recovery trajectories. Although participants were not treatment‐seeking, enrollment in a longitudinal study focusing on alcohol use may reflect a higher baseline motivation to monitor or reduce drinking behaviour. Consequently, individuals who completed the study may have been more motivated to change their alcohol consumption and therefore more likely to achieve spontaneous remission than those who did not complete follow‐up assessments. Although the dropout sample was largely comparable to the continuing sample, differences were observed in a small number of variables including migration background, smoking status and alcohol consumption per weekday. Little's MCAR test indicated that missing data were not completely random, suggesting that selective dropout cannot be fully excluded. As such, differences in baseline characteristics and motivations between completers and non‐completers may have limited the generalizability of the findings to individuals motivated to actively monitor their alcohol use over extended periods. The analytic sample at follow‐up may therefore represent a somewhat selected subgroup, which could have contributed to higher observed remission rates and may limit the generalizability of the identified predictors to the broader AUD population. Nevertheless, most baseline measures, including AUD symptom severity, alcohol consumption patterns and key clinical variables such as stress, anxiety and psychological distress, did not differ between groups, indicating that any resulting bias is likely modest, although reduced sample size may have slightly decreased statistical power relative to the full cohort. In addition to examining group differences at the baseline and at the 1‐year follow‐up, we also analysed follow‐up group differences while adjusting for baseline values. Although data were available at only two time points, these analyses provide an initial indication of temporal dynamics. The included questionnaires varied in their recall periods, with some capturing longer term drinking patterns (e.g., AUDIT) and others reflecting more proximal experiences (e.g., perceived stress). Although this heterogeneity may influence the interpretation of feature importance, it is less likely to have affected predictive performance, as all instruments were validated measures with clearly defined recall periods, ensuring standardization and comparability. Finally, the machine learning analyses yielded moderate predictive performance, indicating that unmeasured variables, external influences or changes occurring during the follow‐up period may have contributed to observed outcomes. These factors were beyond the scope of the present study, which aimed to determine whether baseline characteristics provide sufficient information to identify individuals at higher risk of persistent AUD versus those likely to experience spontaneous remission. Additionally, because AUD criteria were reassessed only at the 1‐year follow‐up, the precise timing of remission could not be determined.

### 4.2 Conclusions

Findings from this longitudinal multi‐site cohort study show that individuals who achieved spontaneous remission exhibited significantly lower AUD severity, reduced alcohol consumption and lower psychological distress, thereby supporting the clinical validity of the remission definition proposed by the NIAAA, which is based on the absence of DSM‐5 AUD criteria except craving. The additional clinical factors identified in the present study therefore extend and refine this definition. Furthermore, the results show that clinical baseline features, especially the AUDIT score, alcohol use measures and AUD severity, can help distinguish individuals who prospectively remit from those who do not. These findings suggest that such features may be useful for recognizing individuals at elevated risk for persistent AUD in the absence of specialized addiction treatment. Future research should explore whether targeted interventions for this group can increase the likelihood of remission.

## Author Contributions

**Judith Zaiser:** conceptualization, methodology, investigation, formal analysis, writing – original draft, writing – review and editing. **Johannes Nitsche:** methodology, formal analysis, writing – original draft, writing – review and editing. **Sabine Hoffmann:** conceptualization, supervision, writing – review and editing. **Sina Vetter:** writing – review and editing. **Nadja Samia Bahr:** writing – review and editing. **Clarissa Grundmann:** writing – review and editing. **Samanda Krasniqi:** writing – review and editing. **Fabian Arntz:** methodology, formal analysis, writing – original draft, writing – review and editing, conceptualization, supervision. **Jens Strehle:** writing – review and editing. **Michael Marxen:** conceptualization, supervision, writing – review and editing. **Sabine Vollstädt‐Klein:** conceptualization, supervision, writing – review and editing. **Michael A. Rapp:** methodology, formal analysis, writing – original draft, writing – review and editing, conceptualization, supervision. **Bernd Lenz:** conceptualization, supervision, writing – review and editing. **Rainer Spanagel:** conceptualization, supervision, writing – review and editing. **Michael Smolka:** conceptualization, supervision, writing – review and editing. **Andreas Heinz:** conceptualization, supervision, writing – review and editing. **Emanuel Schwarz:** conceptualization, methodology, investigation, formal analysis, writing – original draft, writing – review and editing. **Falk Kiefer:** conceptualization, methodology, investigation, formal analysis, writing – original draft, writing – review and editing, supervision. **Patrick Bach:** conceptualization, methodology, investigation, formal analysis, writing – original draft, writing – review and editing.

## Funding

This study was supported by the Deutsche Forschungsgemeinschaft (402170461 ‐ TRR 265) and German Center for Mental Health (DZPG) (01EE2504D).

## Ethics Statement

The study was approved by local Ethics Committees in accordance with relevant ethical guidelines.

## Consent

All participants provided written informed consent according to the Declaration of Helsinki.

## Conflicts of Interest

The authors declare no conflicts of interest.

## Supporting information


**Table S1:** List of questionnaires and variables assessed at the two time points and applied in the machine learning model.
**Table S2:** Details on applied questionnaires.
**Table S3:** Comparison of the drop‐out sample versus the continuing sample at baseline.
**Table S4:** Results of the consistency test of the random forest model.
**Figure S1:** CONSORT study flow chart.
**Figure S2:** Evaluation of model performance I.
**Figure S3:** Evaluation of model performance II.
**Figure S4:** Mean absolute SHAP feature importance.
**Figure S5:** Feature importance stability across age groups.
**Figure S6:** ROC Curve for AUDIT sensitivity.

## Data Availability

Data will be made available upon request.
