# Supplementary material for: Investigating Factors Associated With Spontaneous Remission in Individuals With Alcohol Use Disorder—Results From a Multi‐Site Longitudinal Cohort Study
Source: Addict Biol. 2026 May 10;31(5):e70153. doi: 10.1111/adb.70153 (PMC13158517; doi:10.1111/adb.70153)
Supplement: Supplementary file 1 — Table S1: List of questionnaires and variables assessed at the two time points and applied in the machine learning model. Table S2: Details on applied questionnaires. Table S3: Comparison of the drop‐out sample versus the continuing sample at baseline. Table S4: Results of the consistency test of the random forest model. Figure S1: CONSORT study flow chart. Figure S2: Evaluation of model performance I. Figure S3: Evaluation of model performance II. Figure S4: Mean absolute SHAP feature importance. Figure S5: Feature importance stability across age groups. Figure S6: ROC Curve for AUDIT sensitivity. [file ADB-31-e70153-s001.docx]

**Supplements**

***Investigating factors associated with spontaneous remission in individuals with Alcohol Use Disorder – results from a multi-site longitudinal cohort study***

**Content:**

- **Supplementary Methods**
- Criteria for the identification of alcohol use disorder (AUD).
- Dropouts
- Details on: Classification of remission versus persistence of AUD.
- Consistency test of the random forest model.
- **Supplementary Results**
  - Differences between subthreshold AUD and AUD groups at baseline and at the one-year follow-up, including correction for baseline differences.
  - Results of the SHAP analysis: Classification of remission versus subthreshold AUD versus persistence of AUD.
  - Results of the additional SHAP analysis for feature importance stability across age groups.
  - Results of the consistency test of the random forest model.
  - Results of the sensitivity analysis and the identification of a cut-off score for the AUDIT
- **Supplementary Tables**
  - - **Supplementary Table S1.** List of questionnaires and variables assessed at the two time points and applied in the machine learning model.
    - **Supplementary Table S2.** Details on applied questionnaires.
    - **Supplementary Table S3.** Comparison of the drop-out sample versus the continuing sample at baseline.
    - **Supplementary Table S4.** Results of the consistency test of the random forest model.
- **Supplementary Figures**
  - **Supplementary Figure S1.** CONSORT study flow chart.
  - **Supplementary Figure S2.** Evaluation of model performance I.
  - **Supplementary Figure S3.** Evaluation of model performance II.
  - **Supplementary Figure S4.** Mean absolute SHAP feature importance.
  - **Supplementary Figure S5.** Feature importance stability across age groups.
  - **Supplementary Figure S6.** ROC Curve for AUDIT sensitivity.

**Supplementary Methods**

**Criteria for the identification of alcohol use disorder (AUD).**

The AUD criteria defined in the DSM-5 (1) were considered in the analyses. These criteria are described in detail in the following section. (The numbering is provided for clarity and organizational purposes only and is not official.)

**Criterion 1:**

Larger/ longer: Drinking in larger amounts or over longer periods than intended.

**Criterion 2:**

Cut down: Persistent desire or 3 or more unsuccessful efforts to stop, cut down, or control drinking.

**Criterion 3:**

Time spent: A great deal of time spent in activities necessary to obtain, use, or recover from the effects of drinking.

**Criterion 4:**

Craving: Craving or a strong desire or urge to use alcohol.

**Criterion 5:**

Non-fulfillment of obligations: Recurrent use of alcohol resulting in a failure to fulfill major role obligations at work, school, or home.

**Criterion 6:**

Social/ interpersonal: Continued alcohol use despite having persistent or recurrent (>= 3 times) social or interpersonal problems caused or exacerbated by the effects of alcohol.

**Criterion 7:**

Giving up/ reduction: Important social, occupational, or recreational activities given up or reduced because of drinking.

**Criterion 8:**

Hazardous use: Recurrent alcohol use (>= 3 times) in situations in which it is physically hazardous.

**Criterion 9:**

Physical/ psychological: Continued drinking despite knowledge of having a persistent or recurrent physical or psychological problem that is likely to be caused or exacerbated by drinking.

**Criterion 10:**

Tolerance: Need for markedly increased amounts of alcohol to achieve intoxication or desired effect or a markedly diminished effect with continued use of the same amount of alcohol.

**Criterion 11:**

Withdrawal: The characteristic withdrawal syndrome for alcohol or drinking to relieve or avoid withdrawal symptoms.

**Dropouts**

We collected dropout reasons for n=241 (total dropouts: n=285; missings: n=44) participants. Among these participants, n=48 (19.92%) reported a loss of interest in continued participation, n=64 (26.56%) perceived participation as too time consuming or insufficiently compensated, and n=3 (1.24%) required qualified treatment. In addition, n=29 (12.03%) did not provide a specific reason for discontinuation, n= 64 (26.56%) reported other reasons, and n=33 (13.69%) participants could no longer be contacted and did not provide any information regarding their discontinuation.

Differences between the individuals that continued the study and individuals that dropped out

Participants who completed the study met an average of 4.04 (SD= 1.65) AUD criteria at baseline, whereas participants who discontinued the study met an average of 4.15 (SD= 1.67) AUD criteria. No significant differences were observed between the continuing and dropout samples in age (t=-1.93, p=.054), gender (χ² = 0.27, p=.604), or baseline AUD symptom count (t=0.85, p=.395), nor for the majority of drinking-related variables. However, differences were identified for a small number of variables including migration background and smoking status (for more results of the comparison see **Supplementary Table S3**). To assess the pattern of missing data, Little’s MCAR test was conducted. The test yielded χ² = 150.44, df=92, p<.001, indicating that the missing data were not completely at random.

**Details on: Classification of remission versus persistence of AUD**

To predict group membership at the one-year follow-up across three outcome classes (persistent AUD, subthreshold AUD, and remission), a RF classifier was selected for its ability to model complex non-linear relationships and interactions among predictors. The model incorporated all baseline variables listed in Table S1 as predictors. The RF classifier was implemented with 10,000 trees and balanced class weights to address the pronounced class imbalance in the dataset. Class weights were assigned inversely proportional to class frequencies in the training data, ensuring that minority and majority classes contributed equally to the training process and reduced bias toward the overrepresented class. For reproducibility, a fixed random seed was used. All remaining hyperparameters were retained at their default settings to maintain model simplicity and interpretability.

To evaluate the statistical significance of the model’s predictive performance, we employed a rigorous permutation testing approach to assess whether classification accuracy exceeded chance levels.

Permutation testing was conducted within a stratified 10-fold cross-validation framework. For each fold, class labels in the training set were randomly permuted, after which the RF classifier was trained on the permuted data and evaluated on the corresponding unaltered test set. Model performance was quantified using the area under the receiver operating characteristic curve (AUC-ROC), averaged across folds.

This process was repeated 500 times using a fixed seed to generate a robust null distribution of AUC-ROC values representing model performance under random label assignments. The mean AUC-ROC obtained from the model trained on true labels was then compared with this null distribution. The null hypothesis posited that there was no difference between the performance of the model trained on true labels and models trained on randomly permuted labels. Statistical significance was assessed by calculating an empirical p-value defined as the proportion of permutation runs yielding an AUC-ROC greater than or equal to that of the true-label model, with a correction applied to prevent zero p-values. Mathematically, this was expressed as: p = (N_permutations_exceeding + 1) / (Total_permutations + 1), where N_permutations_exceeding is the number of permutations with AUC-ROC values at least as high as the observed performance. The null hypothesis was rejected at a 95% confidence level if the computed p-value was less than 0.05, indicating that the model’s predictive performance was significantly better than expected by chance.

This comprehensive permutation testing procedure provided a robust assessment of the model’s statistical significance, while accounting for the complexities of multi-class classification and potential overfitting. Feature relevance was first evaluated using Gini feature importance, also known as mean decrease impurity, an intrinsic measure of feature importance in RF models.

Gini importance quantifies the contribution of each feature to reducing weighted impurity across all trees in the ensemble and is directly derived from the optimization criterion used during model training, namely the minimization of Gini impurity at each split. For this analysis, we leveraged the built-in Gini importance calculation provided by the sklearn implementation of RF. To obtain stable estimates of feature importance, Gini importance values were calculated separately for each feature within each fold of the 10-fold cross-validation procedure and subsequently averaged across folds.

In addition, Shapley Additive Explanation (SHAP) values were computed to complement Gini importance by providing both global and local interpretability. Unlike Gini importance, SHAP values allow for an evaluation of feature impacts at the level of the overall as well as individual predictions. For each fold of the 10-fold cross-validation, SHAP values were calculated using the TreeSHAP algorithm, which estimates the marginal contribution of each feature to model predictions by considering all possible feature combinations.

**Consistency test of the random forest model**

To test the consistency of our results and assess potential method dependence, we ran our pipeline with three additional machine learning models from different families; Logistic Regression, linear Support Vector Machines (SVM), and K-Nearest Neighbors (k=3 and k=5). As done for the RF, we assessed model performance (AUC) using 10-fold cross-validation.

**Supplementary Results**

**Differences between subthreshold AUD and AUD groups at baseline and at the one-year follow-up, including correction for baseline differences.**

At baseline: comparisons between the subthreshold AUD and persistent AUD groups revealed lower AUD symptom counts (mean ± SD: 3.4 ± 1.18 vs. 4.48 ± 1.67; p_Subthreshold AUD vs. AUD_<.001), lower AUDIT scores (12.41 ± 4.65 vs. 16.32 ± 4.8; p_Subthreshold AUD vs. AUD_<.001), lower alcohol consumption during the three months prior to baseline (5.14 ± 2.49 vs. 6.73 ± 3.99; p_Subthreshold AUD vs. AUD_=.001) and a lower percentage of heavy drinking days (15.97 ± 20.49 vs. 25.34 ± 25.66; p_Subthreshold AUD vs. AUD_=.004) in the subthreshold AUD group. In addition, individuals with subthreshold AUD exhibited lower comorbid substance use (χ²(2, N=461)=12.02; p_Subthreshold AUD vs. AUD_=.004), lower smoking prevalence (χ²(2, N=443)=11.08, p_Subthreshold AUD vs. AUD_=.006), lower PSS scores (14.12 ± 5.83 vs. 16.44 ± 6.68; p_Subthreshold AUD vs. AUD_=.014), lower ADS scores (8.25 ± 5.22 vs. 11.28 ± 7.3; p_Subthreshold AUD vs. AUD_=.002), lower BSI scores (16.93 ± 14.06 vs. 23.11 ± 19.08; p_Subthreshold AUD vs. AUD_=.021), and lower STAI trait scores (36.06 ± 8.04 vs. 40.28 ± 9.89; p_Subthreshold AUD vs. AUD_=.002) compared with individuals with persistent AUD.

At follow-up, including correcting for baseline differences: after controlling for baseline values, individuals in the subthreshold AUD group showed greater reductions in AUD symptom count (estimated mean ± SE: 1.18 ± 0.13 vs. 3.35 ± 0.07; p_Subthreshold AUD vs. AUD_ <.001), AUDIT scores (11.94 ± 0.44 vs. 14.13 ± 0.27; p_Subthreshold AUD vs. AUD_<.001), and percentage of heavy drinking days (12.28 ± 1.75 vs. 19.47 ± 0.99; p_Subthreshold AUD vs. AUD_=.001) compared with individuals with persistent AUD, mirroring patterns observed in comparisons between remission and persistent AUD. Additionally, the subthreshold AUD demonstrated a greater reduction in BSI (19.59 ± 1.98 vs. 25.18 ± 1.18; p_Subthreshold AUD vs. AUD_=.049), indicating reduced psychological distress.

At follow-up: at the one-year follow-up, individuals with subthreshold AUD continued to differ from those with persistent AUD, showing lower AUD symptom counts (mean ± SD: 1 ± 0 vs. 3.47 ± 1.64; p_Subthreshold AUD vs. AUD_<.001), lower AUDIT scores (10.6 ± 5.05 vs. 15.23 ± 5.21; p_Subthreshold AUD vs. AUD_<.001), and a lower percentage of heavy drinking days (9.41 ± 16.92 vs. 21.43 ± 23.59; p_Subthreshold AUD vs. AUD_=.001), similar to the comparison of remission versus AUD. Additionally, the subthreshold AUD group showed lower scores on clinical scales, including lower PSS score (13.83 ± 5.57 vs. 16.72 ± 6.87; p_Subthreshold AUD vs. AUD_=.004), lower ADS score (9.68 ± 6.62 vs. 13.44 ± 8.85; p_Subthreshold AUD vs. AUD_=.002), and lower BSI score (16.2 ± 16.12 vs. 27.16 ± 24.31; p_Subthreshold AUD vs. AUD_=.001), reflecting lower psychological distress.

**Results of the SHAP analysis: Classification of remission versus subthreshold AUD versus persistence of AUD.**

The SHAP analysis corroborated the findings from the Gini importance analysis, indicating the AUDIT score as the most influential feature for differentiating the persistent AUD group from the two other outcome groups. In addition, the total number of AUD criteria and the percentage of drinking days at baseline were the second and third most influential predictors, respectively, for distinguishing the AUD group from the subthreshold AUD and remission groups. In the SHAP feature importance plots, each bar represents the contribution of a given feature to the prediction of a specific outcome class, independent of the other classes. As illustrated in Supplementary Figure S4, features directly related to alcohol use disorder (e.g., the AUDIT score, sum of AUD symptom count, and percentage of drinking days) exhibited the highest SHAP values, especially for predictions of persistent AUD (**Supplementary** **Figure S4**).

**Results of the additional SHAP analysis for feature importance stability across age groups**

An additional SHAP analysis stratified the sample into age-specific groups (adolescence: 17-32, N=176; early adulthood: 33-49, N=156; adulthood: 50-65, N=95) (2) to account for developmental effects that may differentially influence clinical and social factors (top 10 factors) relevant to the course of AUD and remission. Across all age groups, the AUDIT score is the most salient predictor (see **Supplementary Figure S5**). Comparisons of feature importance ranking across age strata revealed a moderate but non-significant Spearman rank correlation (rho=0.39, p=0.26), indicating largely consistent patterns of predictor relevance across developmental stages.

**Results of the consistency test of the random forest model**

The predictive signal identified by the RF classifier proved highly robust across modeling approaches (see **Supplementary** **Table S4**). Most notably, Logistic Regression achieved a multiclass AUC (0.69), which was effectively identical to the RF model (0.69).
This consistency between a linear model (Logistic Regression) and a non-linear ensemble method (RF) confirms that the predictive signal contained in key variables (e.g., AUDIT scores, age, etc.) is stable and not an artifact of the RF model. RF, Logistic Regression and linear SVM provided superior overall discrimination, as reflected by AUC values across the full probability spectrum.
Based on this performance and its ability to model interactions and non-linear relationships, the RF classifier was retained as the primary model for subsequent analyses, while offering performance equivalent to the linear baseline and support interpretability through SHAP analysis.

**Results of the sensitivity analysis and the identification of a cut-off score for the AUDIT**

Results indicated that the AUDIT score yielded an AUC of 0.724, reflecting moderate discriminative performance. ROC curve analysis (see **Supplementary Figure S6**) identified an optimal cut-off score of >11 on the AUDIT, corresponding to a sensitivity of 0.852, a specificity of 0.492, and a Youden index of 0.344.

**Supplementary Tables**

**Table S1**. List of questionnaires and variables assessed at the two time points (baseline, one-year follow-up) and list of baseline variables that were used as input for the machine learning model.

|  | **ANOVAS** | | **Machine Learning Model** |
| --- | --- | --- | --- |
|  | T0 | FU3 | T0 |
| **Sociodemographical variables** |  |  |  |
| Gender (female/ male) | X |  | X |
| Age (years) | X |  | X |
| Family status (living alone or not) | X |  | X |
| Own children (yes/ no) | X |  | X |
| Migration background (yes/ no) | X |  | X |
| Graduation* | X |  | X |
| Job, last three months (yes/ no) | X |  | X |
| Income (net; <2000€; >=2000€) | X |  | X |
| **Substance use patterns** |  |  |  |
| Sum of met criteria for identification of Alcohol Use Disorder (AUD criteria, DSM-5) | X | X | X |
| Individual AUD criteria, 1-10** (AUD criteria, DSM-5) |  |  | X |
| Alcohol Use Disorder Identification Test (AUDIT) | X | X | X |
| Comorbid substance use (none/ use of at least one further substance) | X |  | X |
| Smoking (yes/ no) | X |  | X |
| Cannabis use last 3 months (yes/ no) | X |  | X |
| **Clinical scales** |  |  |  |
| Perceived Stress Scale (PSS) | X | X | X |
| General Depression Scale (ADS) | X | X | X |
| Brief Symptom Inventory (BSI) | X | X | X |
| State-Trait Anxiety Inventory (STAI trait) | X |  | X |
| Childhood Trauma Screener (CTS) | X |  | X |
| Mental health status (DSM-5) (healthy/ at least one more mental disorder) | X |  | X |
| Depression, current (DSM-5)*** |  |  | X |
| Depression, lifetime (DSM-5) |  |  | X |
| **Alcohol Use** |  |  |  |
| Last 3 months (g alcohol/day) (Quantity Frequency) | X |  | X |
| Typical weekday (g alcohol/day) (Quantity Frequency) | X |  | X |
| Typical weekend (g alcohol/day) (Quantity Frequency) | X |  | X |
| Last drinking day (g alcohol) (Quantity Frequency) | X |  | X |
| Percent drinking days (standard drinks/day) (Quantity Frequency) | X | X | X |
| Percent heavy drinking days (standard drinks/day) (Quantity Frequency) | X | X | X |

*Graduation: Student/ Student of a vocational preparatory, technical, or similar specialized school/ left school without obtaining a lower secondary school diploma/ lower secondary school diploma/ intermediate secondary school diploma/ diploma form a polytechnic secondary school/ entrance qualification for universities of applied sciences/ general university entrance qualification.

**AUD criterion 11, related to withdrawal symptoms, was not included in the analyses, as withdrawal symptoms were an exclusion criterion and thus did not occur in the study sample.

***Depression was included as an additional variable, as it is one of the most common comorbidities in AUD.

Table S2. Details on applied questionnaires.

| Title | Construct | Scale | Recall period | Reference |
| --- | --- | --- | --- | --- |
| Alcohol Use Disorder (AUD criteria, DSM-V) | AUD severity | ordinal | Past 12 months | American Psychiatric Association, 2013 |
|  |  |  |  |  |
| Alcohol Use Disorders Identification Test (AUDIT) | AUD severity | metric | Past 12 months (last two questions refer to lifetime) | Saunders et al., 1993 |
|  |  |  |  |  |
| Perceived Stress Scale (PSS) | Chronic stress | metric | Past month (last 4 weeks) | Cohen et al., 1983 |
| General Depression Scale (ADS) | Depression | metric | Past 7 days | Radloff, 1977 |
| Brief Symptom Inventory (BSI) | Psychological distress | metric | Past 7 days | Derogatis, 1975 |
| Childhood Trauma Screener (CTS) | Childhood trauma | metric | Childhood (before age 18) | Grabe et al., 2012 |
| State-Trait Anxiety Inventory (STAI trait) | Anxiety | metric | General | Spielberger et al., 1970 |
| Quantity Frequency | Alcohol consumption pattern | metric | Past 3 months | Kuitunen-Paul et al., 2017 |

Table S3. Comparison of the drop-out sample versus the continuing sample at baseline.

| Sample |  |  |  |  | |
| --- | --- | --- | --- | --- | --- |
|  | **Continuation**  **(n=462)** | **Drop-out**  **(n=285)** | **Statistics** | | **Significance** |
| *Demographical variables* |  |  |  | |  |
| Gender (female; male) | 176; 286 | 114; 171 | χ² = 0.27 | | p = .604 |
| Age (years) | 37.54 (12.73) | 35.68 (12.85) | t = -1.93 | | p = .054 |
| Family status (living alone or not) | 265; 186 | 146; 90 | χ² = 0.62 | | p = .430 |
| Own children (yes; no) | 150; 301 | 81; 155 | χ² = 0.78 | | p = .780 |
| Migration background (yes; no) | 19; 442 | 10; 36 | χ² = 24.07 | | **p < .001** |
| Job, last three months (yes; no) | 364; 87 | 197; 39 | χ² = 0.79 | | p = .374 |
| Income (<2000€; >=2000€) | 242; 209 | 119; 117 | χ² = 0.65 | | p = .420 |
| *Substance use patterns* |  |  |  | |  |
| AUD criteria | 4.04 (1.65) | 4.15 (1.67) | t = 0.85 | | p = .395 |
| AUDIT | 14.69 (5.14) | 15.88 (5.78) | t = 2.73 | | **p = .009** |
| Comorbid substance use (none; use of at least one further substance) | 405; 56 | 38; 5 | χ² = 0.01 | | p = .920 |
| Smoking (yes; no) | 190; 253 | 122; 108 | χ² = 6.28 | | **p = .012** |
| Cannabis use last 3 months (yes; no) | 116; 346 | 90; 140 | χ² = 14.44 | | **p < .001** |
| *Clinical scales* |  |  |  | |  |
| PSS | 15.62 (6.52) | 16.21 (7) | t = 1.08 | | p = .282 |
| ADS | 10.45 (7.14) | 11.52 (7.77) | t = 1.79 | | p = .073 |
| BSI | 20.77 (18.23) | 22.84 (20.87) | t = 1.25 | | p = .211 |
| STAI Trait | 38.88 (9.86) | 40.25 (10.94) | t = 1.61 | | p = .108 |
| CTS | 7.63 (2.76) | 7.93 (3.29) | t = 1.33 | | p = .183 |
| Comorbid mental illness (healthy; at least one more mental disorder) | 335; 127 | 210; 75 | χ² = 0.12 | | p = .726 |
| *Alcohol drinking* |  |  |  | |  |
| Last 3 months [g alcohol/day] | 6.16 (3.66) | 6.34 (3.41) | t = 0.66 | | p = .512 |
| Typical weekday [g alcohol/day] | 4.22 (3.32) | 3.52 (2.55) | t = -3.04 | | **p = .002** |
| Typical weekend [g alcohol/day] | 7.66 (4.29) | 7.71 (3.95) | t = 0.15 | | p = .882 |
| Last drinking day [g alcohol] | 6.06 (4.73) | 6.75 (4.78) | t = 1.94 | | p = .053 |
| Drinking days (%) | 55.96 (26.6) | 54.59 (24.96) | t = -0.7 | | p = .484 |
| Heavy drinking days (%) | 21.5 (23.95) | 21.1 (20.65) | t = -0.23 | | p = .818 |

Income: with regard to the mean German net income; AUD criteria = Alcohol Use Disorder diagnostic criteria; AUDIT= Alcohol Use Disorders Identification Test; PSS = Perceived Stress Scale; ADS = General Depression Scale (German: Allgemeine Depressionsskala); BSI = Brief Symptom Inventory, STAI trait = State-Trait Anxiety Inventory; CTS = Childhood Trauma Screener;

Note: Gender: None of the participants assigned themselves to “divers”; all variables were received to T0;

Continuous variables: Univariate Analyses of Variance with F-Tests, Mean (STD); Categorical variables: Chi^2^, *χ²*, test or Fisher’s exact test, *Z*

**Table S4.** Results of the consistency test of the random forest model.

| **Model** | **AUC (ovr)**  (Mean/Std) |
| --- | --- |
| Linear SVM (C=1.0) | 0.68 / 0.07 |
| KNN (k=5) | 0.65 / 0.07 |
| KNN (k=3) | 0.63 / 0.06 |
| Logistic Regression | 0.69 / 0.07 |
| **Random Forest** | 0.69 / 0.06 |

Ten fold cross validated AUC (one versus rest) for the RF classifier, compared to other established machine learning algorithms (linear support-vector machine (SVM), K-Nearest Neighbors (KNN), Linear Regression). The results confirm the consistency of the RF classifier’s performance.

*(C* and *k* represent our hyperparameter choices.)

**Supplementary Figures**

**Supplementary Figure S1.** CONSORT study flow chart.

**
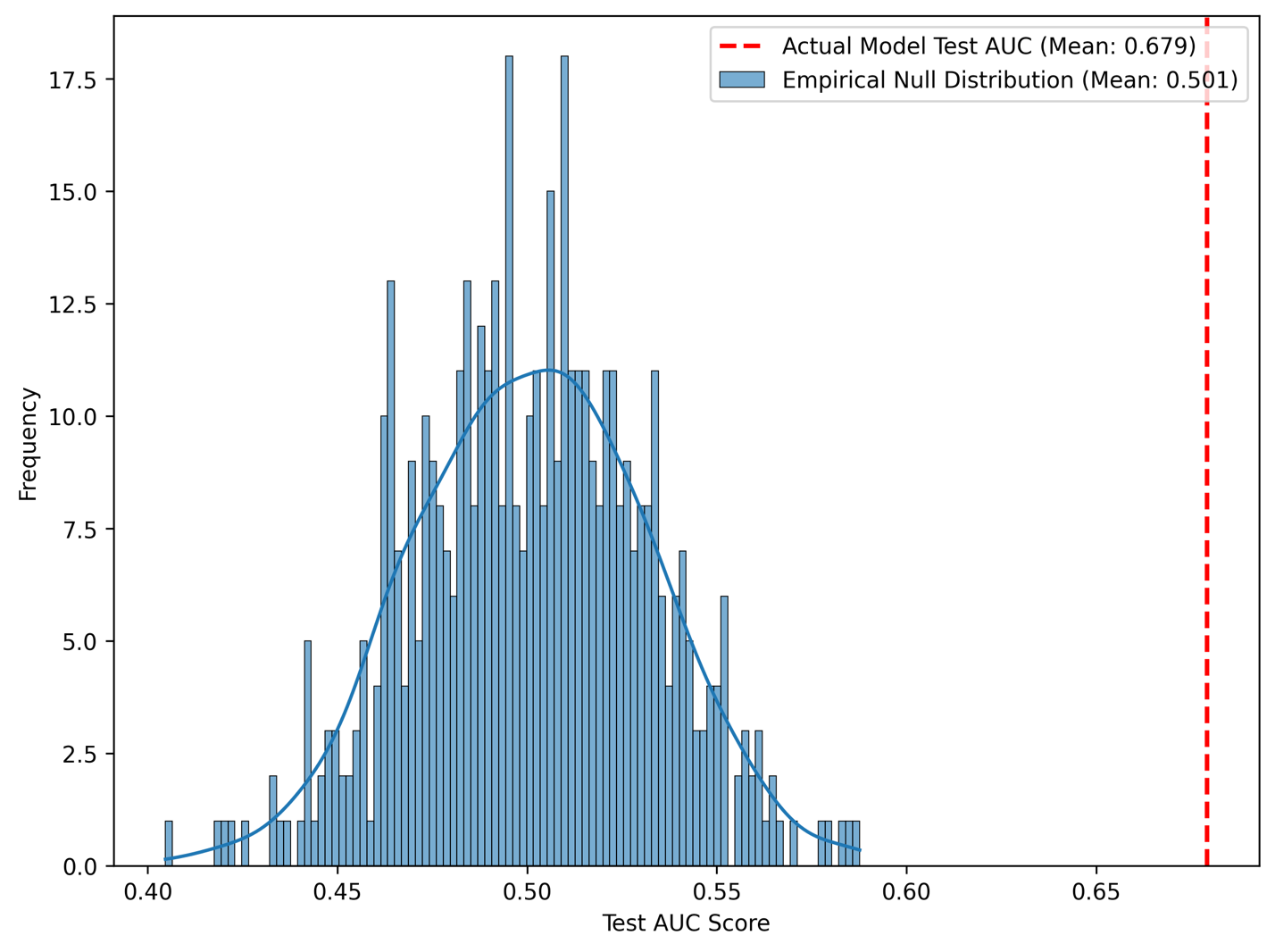
**

**Supplementary Figure S2.** Evaluation of model performance I. Comparison of the empirical null distribution of average test AUC scores (blue histogram with KDE curve) to the actual model's average test AUC score (red dashed line). The null distribution, centered around a mean of 0.50, represents performance under random conditions, while the actual model's AUC of 0.68 is significantly (p= .002) higher, suggesting strong predictive performance beyond chance.

**
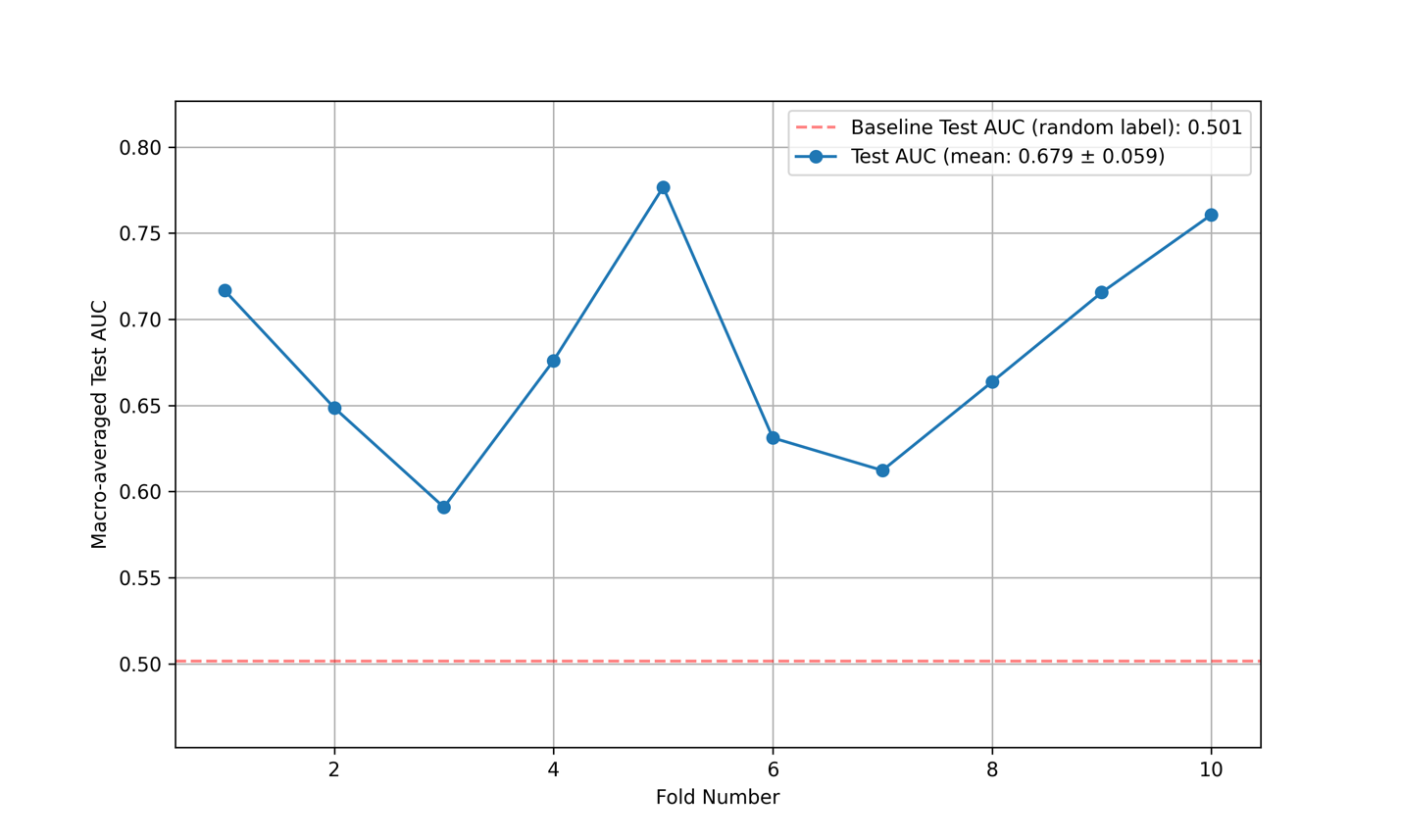
**

**Supplementary Figure S3.** Evaluation of model performance II. Macro-averaged test AUC scores for random forest model across 10 cross-validation folds. The blue dots represent the model's performance in each fold, with a mean AUC of 0.68 ± 0.08. The red dashed line indicates the empirical baseline AUC (0.50) estimated from random predictions.

**
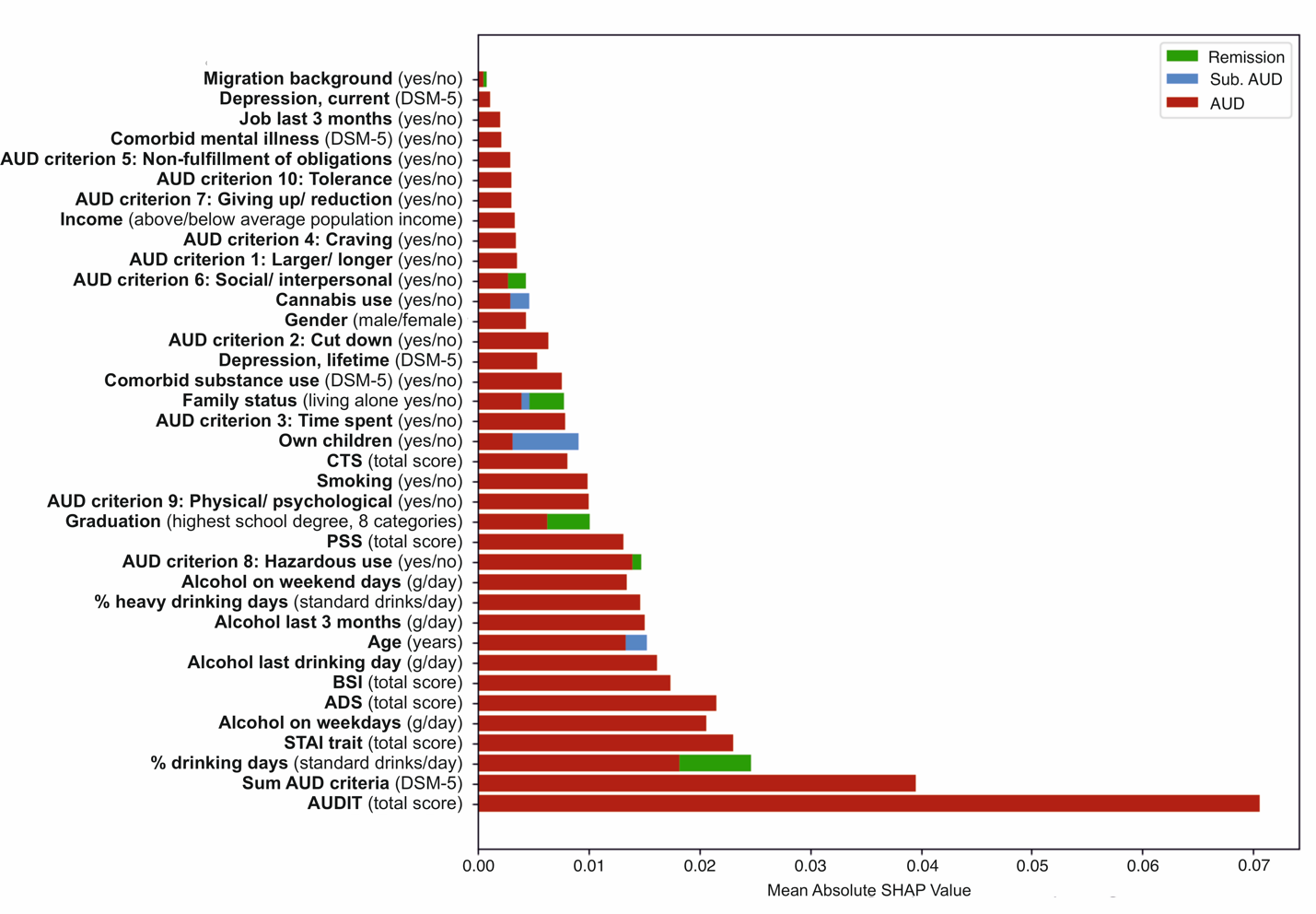
**

**Supplementary Figure S4.** Mean absolute SHAP feature importance. For each feature the individual SHAP value contributions for each class are represented by different colored bars. The features *AUDIT* sum and *the sum of AUD criteria* are most relevant for differentiating AUD from the other groups.

**
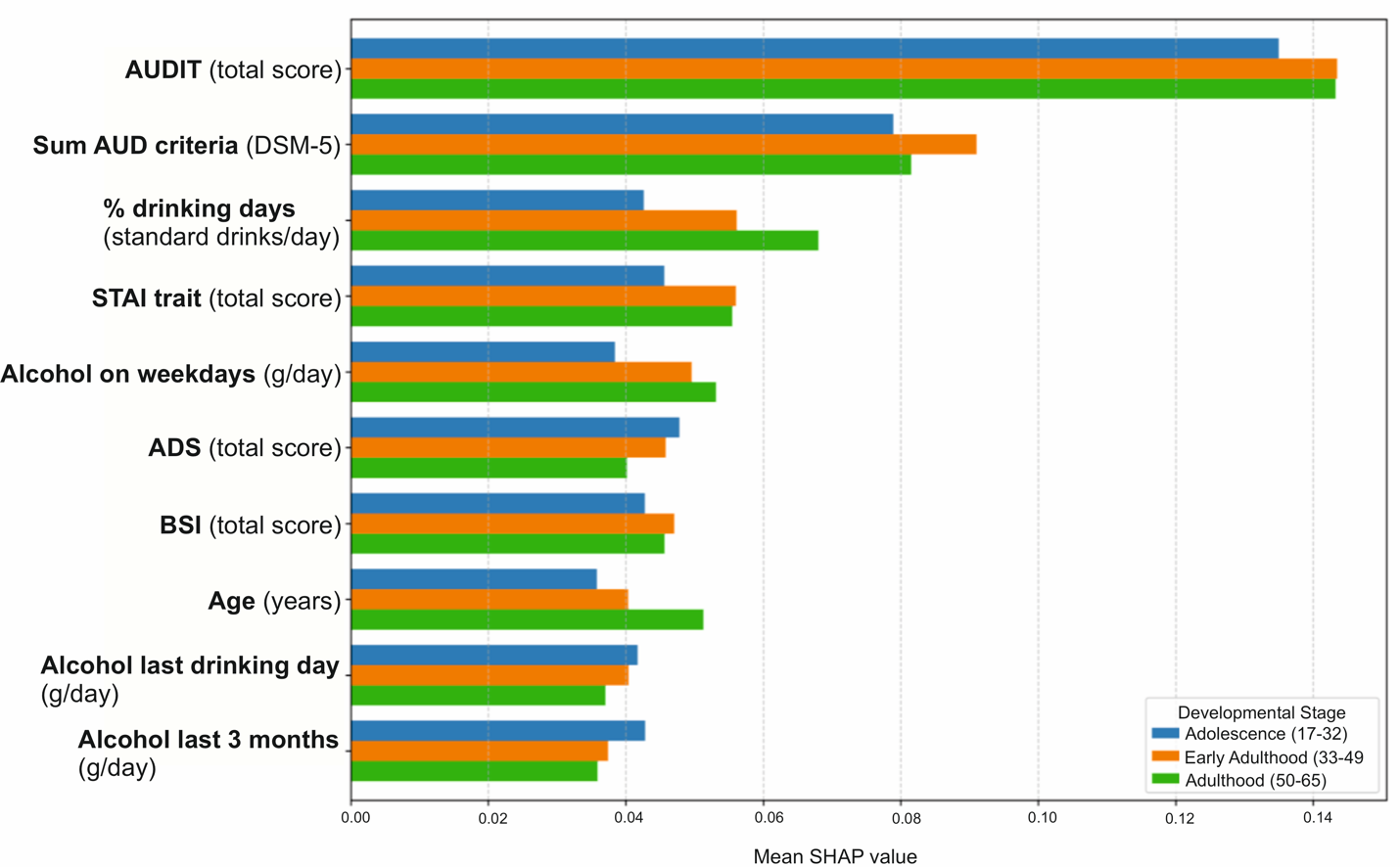
**

**Supplementary Figure S5.** Feature importance stability across age groups.

Spearman correlation of SHAP feature (top 10) rankings was investigated between adolescents (17-32), N=176, early adulthood (33-49), N=156, and adulthood (50-65), N=95. The evidence suggests that the AUDIT sum (and the AUD sum) is the most salient predictor across these developmental stages. Considering all 10 obtained feature rankings, results show non-significant correlations (rho=0.39, p=0.26) across age groups.

**
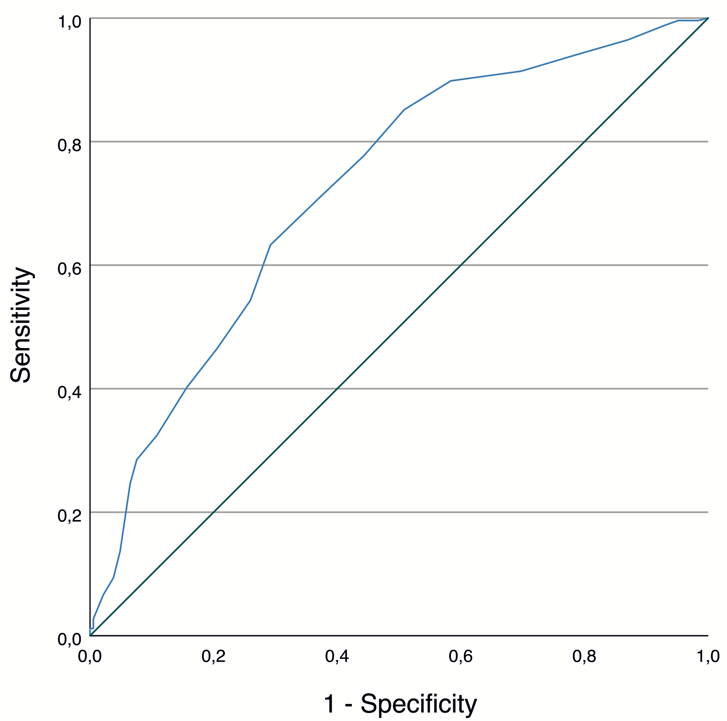
**

**Supplementary Figure S6.** ROC Curve for AUDIT sensitivity**.** The receiver operating characteristic (ROC) curve evaluates the model performance. AUDIT scores of individuals with persistent AUD (AUD) versus non-persistent AUD (subthreshold AUD, remission) are evaluated for sensitivity of classification with an AUC of 0.72 and a Youden-Index of 0.34.

**REFERENCES**

1. American Psychiatric Association, others (2013): Diagnostic and statistical manual of mental disorders (5e éd.). Washington, DC, É. *U: Auteur*.

2. Heinz A, Kiefer F, Smolka MN, Endrass T, Beste C, Beck A, *et al.* (2020): Addiction Research Consortium: Losing and regaining control over drug intake (ReCoDe)—From trajectories to mechanisms and interventions. *Addiction Biology* 25. https://doi.org/10.1111/adb.12866
